# Characterization of the Dynamic Behavior of Neutrophils Following Influenza Vaccination

**DOI:** 10.3389/fimmu.2019.02621

**Published:** 2019-11-20

**Authors:** Diego Ulisse Pizzagalli, Irene Latino, Alain Pulfer, Miguel Palomino-Segura, Tommaso Virgilio, Yagmur Farsakoglu, Rolf Krause, Santiago F. Gonzalez

**Affiliations:** ^1^Institute for Research in Biomedicine, Università della Svizzera italiana, Bellinzona, Switzerland; ^2^Institute of Computational Science, Università della Svizzera italiana, Lugano, Switzerland; ^3^Salk Institute, San Diego, CA, United States

**Keywords:** intravital 2-photon, innate immunity, vaccination, neutrophils, data mining, action recognition, cell actions

## Abstract

Neutrophils are amongst the first cells to respond to inflammation and infection. Although they play a key role in limiting the dissemination of pathogens, the study of their dynamic behavior in immune organs remains elusive. In this work, we characterized *in vivo* the dynamic behavior of neutrophils in the mouse popliteal lymph node (PLN) after influenza vaccination with UV-inactivated virus. To achieve this, we used an image-based systems biology approach to detect the motility patterns of neutrophils and to associate them to distinct actions. We described a prominent and rapid recruitment of neutrophils to the PLN following vaccination, which was dependent on the secretion of the chemokine CXCL1 and the alarmin molecule IL-1α. In addition, we observed that the initial recruitment occurred mainly via high endothelial venules located in the paracortical and interfollicular regions of the PLN. The analysis of the spatial-temporal patterns of neutrophil migration demonstrated that, in the initial stage, the majority of neutrophils displayed a patrolling behavior, followed by the formation of swarms in the subcapsular sinus of the PLN, which were associated with macrophages in this compartment. Finally, we observed using multiple imaging techniques, that neutrophils phagocytize and transport influenza virus particles. These processes might have important implications in the capacity of these cells to present viral antigens.

## Introduction

The innate immune system plays a critical role in protecting the host during the first hours that follow a new insult (1). This process involves complex cell-to-cell and cell-to-pathogen interactions that are essential for the early recognition of the pathogen and the initiation of the adaptive immune response (2). Although several advances have been made in linking the behavior of innate immune cells to the efficiency of the immune response (3), many questions remain open. This is mainly due to the dynamic nature of the aforementioned interaction patterns, which change over time and are distributed in space (4).

The lymph node (LN) has been the preferred organ to investigate *in vivo* the complexity of cell behavior and cell dynamics in relation to immune functions (5). This organ is highly compartmentalized and is composed of specific regions, which facilitate the coordination of the innate and adaptive immune responses. Indeed, the architecture of the LN further promotes the dynamics of immune cell interactions, such as antigen trafficking between macrophages from different regions, which is critical for the final effector response (6–10), and the capture and presentation of antigen by LN resident dendritic cells (DC) (11, 12). The migration of different cell populations to the specific regions of the LN follows a complex balance of chemokine gradients that orchestrate its architecture. For instance, CCL21 and CXCL12 act on the vascular endothelium to promote the recruitment of leukocytes via high endothelial venules (HEV) (13, 14). After the extravasation process, CXCL13 and CCL19-21 direct B and T cells toward the B-cell follicle and the T-cell zone, respectively (15–20).

Among the innate cells that migrate to the LN in inflammatory conditions, neutrophils constitute the first line of defense against pathogens (21, 22). These cells have important immunological functions, such as the secretion of antimicrobial compounds (23), and play a key role in tissue cleaning and remodeling (24). Neutrophils are abundant in the circulation in their mature form and are rapidly recruited from the bone marrow upon inflammation (25, 26) via post-capillary vessels (27, 28) or lymphatics (28–31). Neutrophil recruitment to the site of infection is a highly regulated process that involves the initial secretion of pro-inflammatory factors, released by activated macrophages and DC, which regulate the expression of adhesion molecules from vascular endothelial cells (24, 32). Among the different inflammatory cytokines that have shown to be involved in this process, the interleukin-1 (IL-1) family (33–35) and the tumor necrosis factor (TNF) are some of the best-characterized (29). In addition, many other chemokines and receptors are known to be involved (36).

Once recruited to the inflamed tissue, neutrophils can interact with lymphocytes and antigen-presenting cells (APC) influencing the adaptive immune response (24, 37, 38). This was demonstrated in different inflammatory conditions in which neutrophils released B cell-stimulating molecules, such as BAFF or CD40L (39), or induced T cell proliferation and activation (37, 38, 40). T cell response can be further orchestrated by neutrophils influencing both DC priming and T cell function via NETosis or release of granules (41). Moreover, recent evidence has indicated that neutrophils can cooperate with DC, transporting antigens to the site of T cell activation or acting as APC (21, 42, 43).

While the initial recruitment of neutrophils from blood has been extensively characterized (44), their post-recruitment behavior remains widely unknown. One of the few actions previously described regards the formation of aggregates or swarms (22). This process involves the coordinated migration of cells toward a common target (22, 45, 46). During the formation of swarms, the first neutrophils that are recruited can trigger a cascade of secondary chemoattractants, which amplify the recruitment of other neutrophils in a feed-forward manner (47). The main signals triggering neutrophil influx and swarm formation were associated with tissue injury (48, 49). However, in infection models, other factors such as pathogen-derived compounds (50), or molecules released by dying neutrophils (51) can trigger swarm formation. The role of neutrophil swarms has been linked with microbicidal activity, tissue remodeling, and protection of uninfected tissues (21). However, it is unclear how individual neutrophils behave in the swarming environment. Recent studies using infection models have shown that neutrophil swarm growth is correlated with the removal of subscapular sinus macrophages (22). This suggests an interplay between the two populations and a possible involvement of the resident cell population in the initiation and regulation of the process.

To better investigate the behavior of neutrophils following influenza vaccination, imaging techniques are of paramount importance. Among the available imaging methods, 2-photon intravital microscopy (2P-IVM), allows the long-term observation of cells in tissues of living animals. For this reason, in the last two decades, 2P-IVM has become an essential tool for the observation of immune-related mechanisms *in vivo*, highlighting unprecedented mechanisms related to cell migration and cell-to-cell interaction (5). However, an interdisciplinary approach is required to analyze the imaging data generated by this technique. Indeed, the recently established image-based systems biology approach (52) combines microscopy data with computational methods to describe, quantify, and interpret complex biological processes, from imaging data. This combination of methods allowed for instance to uncover different T cell receptor signaling patterns (53) or different types of migration patterns (54) from the tracks of immune cells.

In this work, we employ a cutting-edge imaging analysis methodology to characterize *in vivo* the dynamics of neutrophil recruitment and their migratory patterns following vaccination with UV-inactivated influenza virus. Thus, we highlight the interaction of early recruited neutrophils with the resident macrophage population involved in antigen capturing. Finally, we report how neutrophil behavior changes over time, using a new mathematical model that maps recurrent motility patterns of neutrophils to biological functions.

## Results

### Neutrophils Are Recruited to the Draining Lymph Node via Blood Following Influenza Vaccination

**Figure 1:**
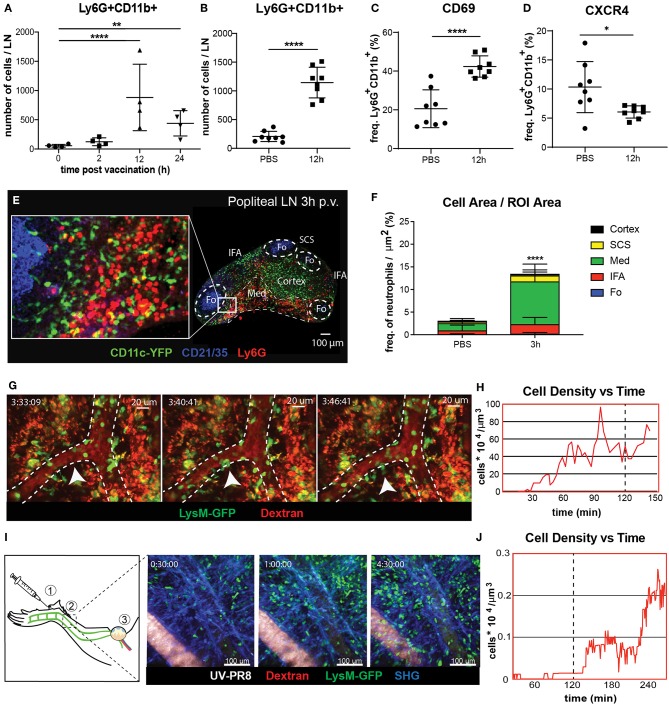
Neutrophil recruitment and distribution into the popliteal lymph node (PLN) after influenza vaccine administration. **(A)** Kinetic of neutrophil recruitment into the PLN during the first 24 h following UV-inactivated influenza virus injection (UV-PR8). **(B)** Flow cytometric analysis showing the recruitment of neutrophils (Ly6G+ CD11b+) at 12 h post-vaccination (p.v.). Percentages of CD69+ **(C)** and CXCR4+ **(D)** cells out of all neutrophils at 12 h p.v. compared with non-vaccinated controls. **(E)** Representative confocal microscopy of LN section showing the distribution of Ly6G+ neutrophils (red) in the LN at 3 h p.v. in a CD11c-YFP animal. **(F)** Quantification plots showing the distribution of neutrophils in the medullary (Med), interfollicular area (IFA), T cell zone (Cortex), follicle (Fo), and subcapsular sinus (SCS) areas of the LN at 3 h p.v.. **(G)** Sequential 2-photon intravital (2P-IV) micrographs showing the recruitment of LysM-GFP neutrophils (green). Blood vessels (red) are labeled by i.v. injection of Rhodamine B isothiocyanate-Dextran. White arrows indicate a hotspot. **(H)** Time series showing increasing density of neutrophils inside high endothelial venules (HEVs) of the PLN following vaccination. (**I**, left) Schematic drawing of the mouse footpad showing the injection site (1), the imaged area (2), and the lymphatic drainage toward the PLN (3). (**I**, right) Sequential 2P-IV micrographs showing the recruitment of LysM-GFP inside the lymphatic vessel in the injection site following UV-PR8 administration. **(J)** Time series showing increasing density of neutrophils inside the draining lymphatic vessel. In all figures, the presented data are representative of at least three independent experiments. Results are given as mean ± SD. ns *p* > 0.05; *p < 0.05; **p < 0.01; ****p < 0.0001.

To study the dynamics of neutrophil recruitment to the popliteal lymph node (PLN) we evaluated the total number of Ly6G+CD11b+ cells by flow cytometry during the first 24 h following footpad administration of influenza vaccine (UV-inactivated influenza virus, strain A/Puerto Rico/8/34). We observed a rapid increase in the number of neutrophils, reaching a peak at 12 h post-vaccination (p.v.) (Figures 1A,B). Moreover, we found that the recruitment coincided with an increase in the expression of the early activation marker CD69 in these cells (Figure 1C). However, we found that, once in the LN, neutrophils downregulate the expression of the chemokine receptor CXCR4, one of the key regulators of leukocyte trafficking (Figure 1D). In addition, we also detect loss and shedding of the receptors CD44, CD62L, and CD49d, which are constitutively expressed in resting neutrophils (Supplementary Figures 1A–D). To better characterize the recruitment process, we performed confocal microscopy and quantified the presence of these cells in different areas of the PLN at 3 h p.v. (Figure 1E). We observed that, at this early time, neutrophils accumulate in the medullary and interfollicular areas (Figure 1F). Next, to evaluate if the recruitment occurs via the blood vessels or the lymphatic system, we monitored *in vivo* the process using intravital 2-photon and electron microscopy. Quantitative analysis of the 2-photon movies acquired in the paracortical blood vessels of LysM-GFP mice showed a prominent increase in the number of neutrophils during the first 2 h p.v. (Figure 1H). In addition, we observed the presence of multiple hotspots (55) in the wall of high endothelial venules (HEVs), suggesting a relevant role of these areas in the observed recruitment (Figure 1G; Supplementary Movie 1). To confirm this observation, we performed electron microscopy in a PLN HEV, which clearly showed neutrophils associated with the blood vessel endothelium as early as 2 h p.v. (Supplementary Figure 1A). Interestingly, intravital imaging of the lymphatic vessels, which drain the area in which the vaccine was administered (Figure 1I), showed a progressive increase in the number of neutrophils inside the lymphatic vessels during the first 4 h p.v. (Figure 1I; Supplementary Movie 2). However, this process appeared to be slower than the recruitment that occurs via blood, as we could not detect any neutrophil in the lumen of the lymphatic vessels during the first 2 h p.v. (Figure 1J).

### The Recruitment of Neutrophils to the LN Involves the Cytokine IL-1α and the Chemokine CXCL1

**Figure 2:**
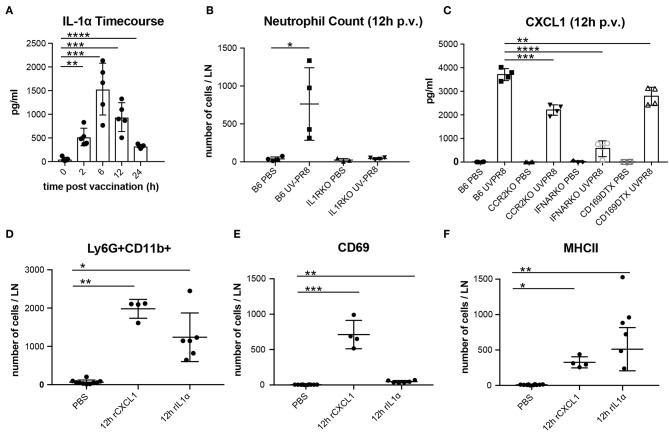
Production of CXCL1 and IL-1α induce recruitment of neutrophils to the PLN in response to vaccination. **(A)** Time course showing the secreted IL-1α in the LN at 0, 2, 6, 12, and 24 h following vaccination. **(B)** Flow cytometric analysis showing the recruitment of neutrophils in the LN in B6 and IL-1R-deficient mice at 12 h p.v.. **(C)** Cytoplex analysis showing the production of CXCL1 in the LN at 12 h p.v. in CCR2 KO, IFNAR KO, and CD169DTR mice compared to B6 controls. Flow cytometric analysis showing the total neutrophils count **(D)**, and the expression of CD69 **(E)**, and MHCII **(F)** after administration of recombinant IL-1α and CXCL1. In all figures, the presented data are representative of at least three independent experiments. Results are given as mean ± SD. ns *p* > 0.05; *p < 0.05; **p < 0.01; ***p < 0.001; ****p < 0.0001.

In a previous study, we observed that the necrotic death of the LN macrophages after the administration of influenza vaccine was followed by the release of the potent inflammatory cytokine IL-1α (6). In this work, we confirmed that IL-1α reaches an early peak (6 h) following vaccination and returns to basal levels at 24 h p.v. (Figure 2A). Moreover, we found that IL-1RKO animals show a significant inhibition of neutrophil recruitment in the PLN (Figure 2B). To confirm that IL-1α was involved in neutrophil recruitment, we injected a dose of 1 μg of recombinant IL-1α in the mouse footpad, and observed that the injection of this cytokine alone was able to induce the recruitment of neutrophil in the draining PLN at 12 h p.v. (Figure 2D). In a previous work, we also observed that influenza vaccination induced a fast increase of the chemokine CXCL1 (6), a well-known inducer of neutrophil recruitment (56). Therefore, to investigate whether the production of this chemokine was associated with LN macrophages, we measured the secretion of CXCL1 in animals in which LN macrophages (CD169-DTR + Diphtheria toxin) or monocytes (CCR2KO) had been depleted. We observed that, in both cases, the levels of CXCL1 were significantly reduced at 12 h p.v. compared to the control group (Figure 2C). However, CXCL1 levels were not completely abrogated. Interestingly, we also observed that the type-I interferon response following vaccination was necessary for the secretion of this chemokine, as IFNARKO animals showed a prominent inhibition of the levels of CXCL1 at 12 h p.v. (Figure 2C). Moreover, we also observed that IFNARKO animals display lower number of resident macrophages (Supplementary Figures 1H,I), while CCR2KO did not showed any difference (Supplementary Figure 1G). However, DTX-treated CD169-DTR mice exhibited a complete depletion of LN macrophages after the administration of the toxin (Supplementary Figure 1F). Besides, footpad administration of recombinant CXCL1 (0.5 μg/fp) alone was able to induce a significant recruitment of neutrophils in the popliteal LN (Figure 2D). However, we observed significant differences in the effect that both molecules had on the activation of neutrophils. Recombinant CXCL1, but not IL-1α, was able to induce the expression of the early activation marker CD69 in the recruited neutrophils (Figure 2E). However, neutrophils recruited after IL-1α administration showed higher levels of MHC II compared to the ones recruited following treatment with CXCL1 (Figure 2F).

### Neutrophils Phagocytize and Transport Influenza Virus

**Figure 3:**
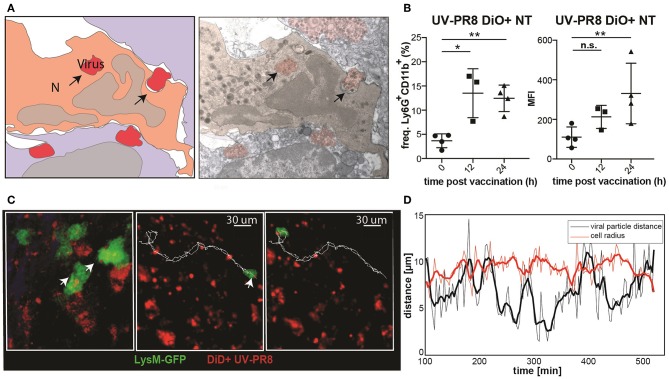
Neutrophils phagocyte and transport influenza virus. **(A)** Schematic drawing (left) of an electron micrograph (right) showing neutrophils in the SCS phagocytizing UV-inactivated influenza virus at 2 h p.v.. **(B)** Flow cytometric analysis indicating the percentage of neutrophils positive for influenza vaccine (DiO labeled, left) and the corresponding MFI analysis (right). **(C)** Intravital 2-photon micrograph showing a LysM-GFP neutrophil (green) phagocytizing DiD labeled UV-PR8 (red) (left). Intravital 2-photon timelapse showing the correlation between the tracks from a UVPR8-positive vesicle and the neutrophil (right). **(D)** Distance of the viral particles from the cell centroid (black line), compared to the cell radius (red line). Results are given as mean ± SD. ns *p* > 0.05; *p < 0.05; **p < 0.01.

To examine the capacity of neutrophils to phagocytize UV-inactivated influenza virus (UV-PR8), we performed electron microscopy. Indeed, results showed that a number of neutrophils phagocytized necrotic vesicles containing the UV-PR8 particles (Figure 3A, Supplementary Figure 1J). To quantify the percentage of neutrophils that phagocytized the virus, we labeled inactivated influenza virus with the lipophilic dye DiO and performed flow cytometric analysis. We found that 15% of the neutrophils were positive at 12 h p.v. (Figure 3B). Finally, to assess the capacity of neutrophils to transport phagocytized influenza particles, we performed 2P-IVM in LysM-GFP mice. The dual tracking of DiD-UV-PR8 and LysM-GFP neutrophils confirmed that, after phagocytosis, neutrophils were able to transport the phagocytized virus within the LN during 7 h (Figure 3C, representative track, Supplementary Movie 3). A volumetric reconstruction further confirmed that viral particles were internalized, with a distance from the cell centroid smaller than the cell radius (Figure 3D).

### Neutrophils Change Their Motility Soon After Being Recruited to the LN

**Figure 4:**
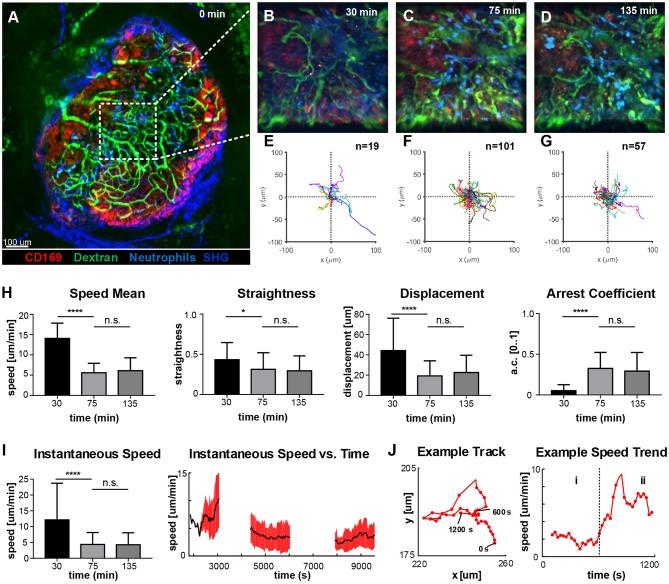
Time-course of neutrophil motility. **(A)**. Low-magnification 2P-IVM micrograph showing the structure of the PLN including vascular network (Fluorescein isothiocyanate-Dextran), CD169+ macrophages (red), collagen scaffold (Second Harmonic Generation–blue) and neutrophils (light-blue) adoptively transferred from a CK6/ECFP animal. Image was acquired immediately after vaccine injection. **(B–D)** Snapshots of representative LN areas at different time points. **(E–G)** Plots of cell tracks with common origin showing four preferential migratory directions at 30 min, more equally distributed directionalities at 75 min and a tendency toward the 1st and 4th quadrants at 135 min. The number of tracks is 19, 101, 57, respectively. **(H)** Cell motility quantification using tracks mean speed, straightness, displacement, and arrest coefficient. **(I)** Boxplots of Instantaneous speed (left) exhibiting high variance at early time points. Instantaneous speed time series (right) showing distinct trends at different time points. **(J)** Representative track (left) acquired at 135 min and the associated time series of the instantaneous speed (right). The same cell exhibits two different trends characterized by low speed (i) and high speed (ii). Results are given as mean ± SD. ns *p* > 0.05; *p < 0.05; ***p < 0.001; ****p < 0.0001.

To identify the areas of the LN with higher activity, we acquired low-magnification 2P-IVM movies (Figure 4A) in a non-fluorescent recipient animal by adoptively transferring neutrophils from a CK6/ECFP donor. Moreover, to visualize the vasculature and the LN macrophages, we administered fluorescein isothiocyanate-dextran (200 kDa) and CD169-PE antibody, respectively. Initially, we imaged an area located in the paracortex of the LN (Figure 4A, dashed line), characterized by a high vascularization and active recruitment of neutrophils. Representative snapshots of the acquired movies are shown in Figures 4B–D, while the tracks of the analyzed cells are plotted in Figures 4E–G. A qualitative analysis of cell migration showed that at 30 min p.v. neutrophils generated long tracks in four main directions, which were associated with their movement inside the blood vessels (Figure 4E; Supplementary Movie 4). Once outside the blood vessel (75 min p.v.), neutrophils did not follow any preferential direction (Figure 4F; Supplementary Movie 4), while at later time points (135 min p.v.) they displayed a collective migration directed toward an area in which cells started to cluster (Figure 4G; Supplementary Movie 4). Next, we quantified cell migration by computing measures based on entire tracks (Figure 4H). We observed a significant change in neutrophil motility occurring within 30–75 min p.v. Indeed, neutrophils at 30 min p.v. were faster, more directional and traveled longer distances with a lower arrest coefficient, compared to later time points. However, no significant difference was observed between 75 and 135 min. These findings, using track-based measures, confirmed a change in the overall motility only after recruitment. Nevertheless, the analysis of the instantaneous speed of neutrophils showed a high variance (Figure 4I, left), which was associated with a variable mean over time (Figure 4I, right, black line). The observed variability in speed arises from both the differences between distinct cells at the changes of speed that a single neutrophil undergoes over time. An example is provided in Figure 4J, where the track of a neutrophil (left) and the plot of the instantaneous speed (right) over time are shown. This example shows the transition between two distinct behaviors that are characterized by low speed and high speed, respectively.

### Neutrophils Perform Different Actions Over Time

**Figure 5:**
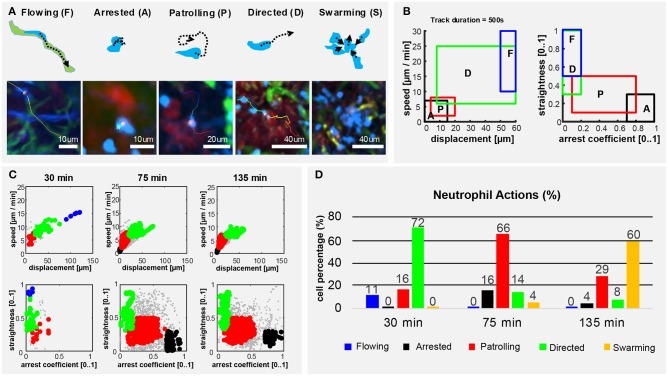
Neutrophils exhibit different actions in the early response to vaccination. **(A)** Graphic representation (up) and 2P-IVM micrographs showing different neutrophil actions in response to influenza vaccination: flowing “F” neutrophils moving inside blood vessels, with long and straight tracks following the vessel structure. Arrested neutrophils “A” exhibit confined tracks and short displacement. Patrolling neutrophils “P” have long, non-straight tracks associated with extensive tissue monitoring. Directed neutrophils “D” exhibit straight tracks with lower migration speed compared to flowing neutrophils. Swarming neutrophils (S) form cell aggregates with high density and large volume. **(B)** Gating strategies used to define and identify the actions according to cell speed, displacement, straightness, and arrest coefficient on track fragments of fixed length (500 s). **(C)** Classification of track fragments at different time intervals (arrested: black, patrolling: red, directed: green, flowing: blue, unspecified: gray). **(D)** Time-course plot showing the percentage of cells performing each of the actions over time, according to our gating strategy.

To describe the long-term and time-varying behavior of each individual neutrophil, we defined, according to previous studies (45, 57–59), five distinct cellular actions based on the motility patterns visually identifiable in the videos (Figure 5A). We named them flowing, arrested, patrolling, directed migration and swarming (Figure 5A). To detect these actions from imaging data, we divided the track of each neutrophil into multiple fragments (tracklets). Then, we computed morphological and motility measures on each of them. By defining a gating strategy on these measures, each tracklet was associated with an action (Figure 5B). Considering each tracklet as a data-point, the proposed gating strategy identified five distinct populations corresponding to the different actions (Figure 5C). Following this analysis, we observed that neutrophils perform different actions at different time points (Figure 5D). At homeostasis, neutrophils were mostly flowing. Hence, circulating within blood vessels with high speed and directionality (Supplementary Figure 2; Supplementary Movie 8). Then, during the first 30 min p.v. neutrophils were mostly associated with capillaries, displaying both flowing and directed migration. This behavior changed when neutrophils started to migrate within the LN (30–75 min p.v.), displaying primarily patrolling, directed, and arrested behaviors. Other neutrophils exhibited a temporarily directed migration. Finally, at 2 h p.v., cluster formation was the predominant neutrophil behavior. This was associated with temporarily directed migration of neutrophils either toward a swarm under formation or from a previously formed swarm to another target. In addition, a small population of neutrophils was arrested (Figure 5D). These results confirmed that the time-varying motility of neutrophils can be represented as a sequence of distinct actions which, in turn, are associated with distinct biological processes.

### Following Vaccination Neutrophils Form Swarms in the SCS Associated With SCS Macrophages (SSM)

**Figure 6:**
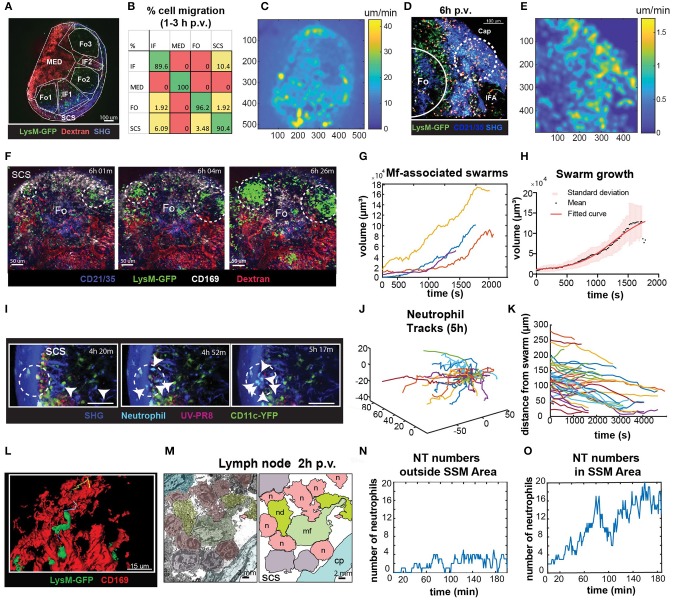
Characterization of neutrophils swarm formation in the PLN after influenza vaccination. **(A)** Low magnification 2P-IVM micrograph showing the PLN of a LysM-GFP animal at 3 h p.v. Seven different areas are identified according to their anatomical structure and cell populations: SCS, follicles (Fo1-3), interfollicular areas (IF1-2), and medullary area (MED). **(B)** Transition matrix showing the migration of cells across different areas. The value at the i-th row and j-th column refers to the percentage of cells that migrated from the i-th to the j-th area. Values on the diagonal refer to the percentage of cells that remained in the same area. **(C)** Pixel velocity heatmap, showing hotspots with high motility. **(D)** 2P-IVM micrograph showing the IF and SCS areas at 6 h p.v. Cap refers to the collagen capsule. Dashed lines indicate hotspots toward which cells migrate with high motility. **(E)** Pixel velocity heatmap showing hotspots with high motility (yellow) **(E)**. Dashed lines indicate hotspots toward which cells migrate with high motility. **(F)** Sequence of micrographs at 6 h p.v. showing the formation of multiple LysM-GFP neutrophils swarms in the SCS in close proximity to CD169+ macrophages (white) **(G)** Swarm volumes over time observed at 6 h p.v.. Volumetric data built according to fluorescence intensity thresholding. **(H)** Sigmoid fit to previous swarm volume (*R* = 0.652) showing their averaged growth phase. **(I)** Sequence of micrographs with 25x magnification in the SCS showing CFP-expressing neutrophils initiating a swarm at 5 h p.v. **(J)** Trajectories of 5 h p.v. swarm plotted from the same origin **(K)** Time-course distances from the swarm center at 5 h p.v. **(L)** High magnification 3D reconstruction of LysM-GFP neutrophils interacting with CD169 macrophage network (red) in the SCS region. **(M)** Colored transmission electron micrograph (left) and schematic drawing (right) showing the interaction of different neutrophils (n) with a macrophage (mf) in the SCS of the LN at 2 h p.v.. **(N,O)** Number of neutrophils vs. time showing an oscillatory trend in an area without macrophages **(N)**, and an increasing trend indicative of swarming in an area associated with macrophages **(O)**.

To identify the areas in the LN with high cell motility we performed low magnification (10X) 2P-IVM, which allowed the visualization of the whole organ (Figure 6A; Supplementary Movie 5). Cells were tracked for a period of 30 min and the percentage of cells migrating from one region to another was computed. We found that at early time p.v. most of the neutrophil migration occurred between the interfollicular (IF) and the SCS areas (Figure 6B). To evaluate the presence of areas associated with high neutrophil motility, we measured the average pixel velocity by optical flow, a computer vision method that does not require cell tracking (60). The results showed the presence of hotspots with high motility, which are depicted as lighter areas in the pixel velocity heat map (Figure 6C). These hotspots were localized in the SCS and IF area. Interestingly, the hotspots were associated with the regions where swarms were formed (Figures 6D,E).

To fully characterize neutrophils swarming behavior, we recorded movies in the SCS region of the LN using different magnifications. Low magnification movies from LysM-GFP mice showed that neutrophils formed large and multiple swarms in association with regions enriched with macrophages (Figure 6F; Supplementary Movie 6). Such swarms grew in size over time (Figure 6G). Furthermore, neutrophils involved in the swarms often changed their directionality from one cluster to another (Figure 6F). In mathematical terms, the average dynamic of the observed swarms was best described by a sigmoidal function (Figure 6H), suggesting that swarm formation undergoes an initial steady state, a growing phase and a plateau before its resolution. Moreover, we observed that the decreasing swarms showed a resolution period of ~30 min.

To better visualize individual neutrophil behavior, as well as possible interactions with resident macrophages, we acquired high-magnification videos starting from 5 to 7 h p.v. These videos confirmed that swarm formation occurs in proximity to SSM (Figure 6I; Supplementary Movie 7), with highly directed and skewed trajectories (Figure 6J), and the majority of the cells approaching the center of the swarm (Figure 6K). Moreover, using high-magnification 3D reconstruction and EM, we confirmed that neutrophils were located in close proximity to SSM clusters (Figures 6L,M). To investigate the involvement of SSM in the formation of swarms we quantified the accumulation of neutrophils in areas proximal or distal to SSM. Interestingly, the number of neutrophils fluctuated over time outside the SCS area (Figure 6N). By contrast, it constantly increased in areas rich in macrophages (Figure 6O) exhibiting the sigmoidal growth rate observed during swarm formation.

## Discussion

In this study, we investigated *in vivo* the behavior of neutrophils that are recruited to the draining LN following influenza vaccine administration. Neutrophil recruitment has been previously described in several infection models (22, 30, 44, 51, 61). However, despite the critical role of these cells in pathogen clearance and the initiation of the inflammatory response, their specific behavior upon vaccination remains poorly studied. In this work, we observed a rapid recruitment of activated and mature neutrophils in response to vaccination with an inactivated influenza virus. Although the role of neutrophils against influenza has been extensively studied in the lung (62), this is the first time that their behavior is characterized in the draining LN in the early response to influenza vaccine administration.

The way neutrophils enter the LN remains controversial. Different studies have suggested that neutrophils get recruited mainly via the HEV (44, 48). However, other authors have stated that neutrophils access the LN mainly via the lymphatic vessels (29–31). Our data suggested that influenza vaccination induces an initial recruitment of neutrophils via HEV, followed by a minor, secondary wave through the lymphatics that drain directly from the injection site to the sentinel LN. It is tempting to speculate that the type of recruitment might influence the function of the recruited cells. However, future experiments need to be performed to study differences in the behavior and the function of neutrophils that arrive through different routes. In addition, we found that a percentage of neutrophils downregulates the expression of CXCR4, a marker known to be involved in neutrophil mobilization from the bone marrow and trafficking through the circulation to the site of inflammation (63, 64). Moreover, consistently with neutrophils activation, we detected a downregulation of multiple cell surface receptors linked with leucocyte trafficking and accumulation (65, 66). By contrast, these receptors were expressed at high levels in resting neutrophils (67).

Neutrophil localization within the LN after pathogen challenge is tightly linked to their specific function. In a previous study, we observed that macrophages, located within the subcapsular sinus (SCS) and the medullary area of the LN, capture and retain influenza virus following vaccination. Interestingly, in both the areas, macrophages undergo necrosis-like cell death after viral capture that leads to their progressive decline (6). Our study demonstrated that neutrophils migrate toward the SCS progressively, probably in response to chemoatactic signals released by the necrotic macrophages, as suggested by other models based on infection (22, 48). Among the different signals released by the necrotic macrophages, IL-1α is one of the most potent (6). In this work, we have demonstrated that IL-1α and its receptor IL-1R are involved in the initial recruitment of neutrophils to the LN. Other authors have previously confirmed the role of IL-1β and IL-1R in the recruitment of neutrophils to the infection site (33, 68, 69). However, we could not observe any significant secretion of IL-1β or activation of the inflammasome pathway in the LN following influenza vaccine administration (6). Therefore, we can conclude that the observed absence of neutrophil recruitment in the IL-1R-defective mice was associated to IL-1α released by macrophages.

Moreover, we confirmed that CXCL1, a mouse homolog of human IL-8, was also involved in the recruitment of neutrophils to the LN. The source of this chemokine needs to be further investigated but we speculate that DC and activated macrophages could be the main producers (70, 71). Indeed, the secretion of CXCL1 was almost abolished in mice lacking type-I interferon (IFN) receptors, suggesting an important role of LN macrophages and DC, the main producers of IFN, in this process. Thus, absence of IFN-I signaling in IFNARKO mice partially reduce the total number of CD169+ macrophages compared to the control, although it did not significantly impair the number of medullary macrophages. However, the specific elimination of macrophages reduced only partially the expression of CXCL1. Interestingly, our findings demonstrated that subcutaneous administration of IL1-α and CXCL1 induced the mobilization of neutrophils toward the LN. Nevertheless, we showed that CXCL1 induced the expression of CD69, a marker associated with the early activation, while IL-1α administration increased the expression of MHCII, suggesting a potential role of neutrophils in antigen presentation, as previously described in other models (40, 72). Neutrophils are sensitive to a vast array of chemoattractants that regulate their migration and infiltration to inflamed tissues. Indeed, Chou et al. (73), previously described this process as a cascade that require a multitude of chemokines, such as MIP-1α, MIP-1β, and MIP-2. Therefore, will be of interest to explore the complex nature of signals that mediate neutrophils recruitment post-influenza vaccination.

In the early phases of recruitment, we found that neutrophils exhibited significant differences, resulting in reduced speed, directionality, and displacement, while increased arrest coefficient. These findings suggest that the recruited neutrophils actively migrate and increase cell-to-cell interactions. Previous studies have associated the increase in the arresting of neutrophils with the oxidative burst (20) in which reactive oxygen species are generated. Antigen presentation might also influence the speed of neutrophils. Indeed, other authors have reported that neutrophils can serve as antigen presenting cells (APC) during influenza infection in mice (2, 74, 75). In support of the notion that neutrophils might act as APC in the context of influenza vaccination, we observed that they actively phagocytize influenza particles, which were previously associated with necrotic macrophages. Moreover, we could observe an increase in the expression of MHCII in these cells after exposure to IL-1α, which is released by the dying macrophages (6). However, it is not clear if the APC function of neutrophils might occur in the LN or, as suggested by other authors (76), in other immune-relevant organs, such as the spleen. In support of the former, we observed that neutrophils are able to transport influenza particles for long distances in the LN (77). This suggests that neutrophils can carry viral particles to specific areas as described for other infection models (78–80). Therefore the potential capacity of the neutrophils to transport viral particles to other organs as well as the capability of these cells to function as APC need to be further investigated.

Our findings supported that neutrophil behavior is a dynamic process, with significant differences observed already within the first 3 h p.v. These findings were in agreement with previous *in vitro* studies in which the motility of neutrophils was found to change within minutes in response to both external (i.e., chemical gradients) and internal factors (i.e., directional memory) (81).

Among the different actions that occur within the first few hours after vaccination, we identified the formation of swarms, which is a process previously associated with tissue injury (49, 51). In this study, we showed that neutrophils swarms are formed in the SCS, co-localizing with the resident SSM population. The characterization of swarm dynamics showed consistent growth rates, suggesting that they are comparable to smaller transient swarms observed in other infection models (22). Regarding the factors that generate this behavior, tissue injury, neutrophil secondary cell death, and the release of the chemoattractant LTB4 have been previously proposed as triggers of swarm formation (51). Although LTB4 is mainly secreted by neutrophils, macrophages can also produce this molecule (51). These observations, along with our *in vivo* evidence of swarms association with SSM, suggest a close association between the two populations. In previous studies, swarm formation in the SCS was linked with the removal of resident SSM (24). We speculate that macrophage death contributes to the initiation, amplification, and stabilization of neutrophil swarming and recruitment via the release of different chemoattractants, such as IL-1α. However, the redundancy in the recruitment process of neutrophils, with the involvement of more than 30 chemokine receptors (36), makes the effect of a single molecule difficult to be distinguished from other cues that regulate neutrophil chemotaxis.

Regarding the swarm dynamics, a direct correlation between swarm size and tissue injury severity has previously been shown (82). Furthermore, the number of neutrophil secondary death is also proportional to the swarm size (51). It would be compelling to determine whether influenza vaccination induces the death of neutrophils in a way similar to the previously described macrophage death (6).

The quantification of the spatio-temporal migration and interaction patterns of cells from 2P-IVM data presents specific challenges. These arise from the difficulties both in cell tracking and in describing a complex biological system by means of numerical values. The difficulties in cell-tracking, arise from both the textureless appearance and the complex biomechanical properties of neutrophils, including high plasticity and formation of contacts. These problems are amplified by the high number of cells that need to be tracked. To facilitate individual tracking, the number of fluorescently-labeled cells can be reduced by performing an adoptive transfer of a limited number of fluorescently labeled cell to a non-fluorescent recipient animal. This justifies the differences in cell number between the experimental setup using LysM-GFP transgenic model or the adoptive transfer of CFP-neutrophils into wild type animals prior to imaging shown in Figures 1, 6, respectively.

In this study, we described an alternative way to analyze cell motility in 2P-IVM videos when single cells cannot be tracked. Indeed, by computing pixel velocity, we identified the areas in the LN, called hotspots, in which cells were more active. The advantage of pixel-based measures with respect to track-based measures is that neither manual nor automatic single-cell tracking is required. Therefore, this allows the analysis of videos with a high number of cells.

The quantification of neutrophil behavior from 2P-IVM data is further challenged by the lack of mathematical models that make their motility patterns interpretable. Although optical probes can be used to transform a biological function into a light signal, their application for 2P-IVM remains challenging (83).

To address this issue, we developed a new method of analysis that brings two main advantages with respect to the previously used methods: capturing the time-varying motility of cells and making results interpretable. Indeed, the available methods to assess cell motility can lose information during the averaging process (81). Additionally, although several measures of cell motility were defined (84), the connection of their values to a biological meaning remains to be addressed by the investigator. When applied to our data, the standard track-based measures did not capture significant differences in neutrophil behavior. However, the new method of analysis, proposed in Figure 5, identified distinct actions, which changed over time, indicating clear differences in the functions of the analyzed cells. Although in this study we defined five distinct actions of neutrophils, alternative actions could be also defined in future studies, such as apoptosis or NETosis (78), amongst others.

The proposed method allowed to perform a dynamic *in situ* cytometric analysis as proposed in previous studies (53, 83) where distinct phenotypes of cells were identified in 2P-IVM data. However, a set of gates correlating phenotypes to actions were not defined previously. By contrast, our action-based model allowed to define a precise set of gates to interpret the results. It would be compelling to define an extended list of actions that neutrophils can perform or automatically unravel populations of cells expressing distinct phenotypes using data mining methods such as clustering algorithms (85, 86). Additionally, advanced computer vision methods can be applied to detect more complex behaviors, both on shorter and longer periods of time considering other parameters such as cell morphology, context, and space-time structures. This is in line with recent works that aim to recognize cellular motion phenotypes in *in vitro* cultures (87) or human actions using deep machine learning methods (88).

In conclusion, to analyze the complex dynamics of neutrophils in intravital imaging data, an interdisciplinary effort is required. By combining different imaging methodologies, molecular techniques, and pattern recognition methods, we identified distinct behaviors of neutrophils in the early response to influenza vaccination. These behaviors are the expression, of the biological mechanisms that follow influenza vaccination. In addition, we identified an interaction between neutrophil and macrophages, which might be important in terms of the capacity of the former to capture and present antigen.

## Methods

### Mice

All animals were bred in-house or acquired from Janvier labs (C57BL/6). Mice were maintained under specific pathogen-free conditions at the Institute for Research in Biomedicine, Bellinzona and used in accordance with the Swiss Federal Veterinary Office guidelines. The following transgenic mice were used: LysM-GFP (89), IL-1R KO (90), TLR3 KO, Myd88 KO (91), CCR2 KO, IFNAR KO (92), CD169DTR (93), CD11c-YFP (94), CK6/ECFP (95), UBC-GFP (96). All strains were on C57BL/6 background. All animal experiments were performed in accordance with the Swiss Federal Veterinary Office guidelines and authorized by the relevant institutional committee (Commissione cantonale per gli esperimenti sugli animali, Ticino) of the Cantonal Veterinary with authorization numbers TI28/17, TI02/14, and TI07/13.

### Virus Production Inactivation and Labeling

Influenza virus strain A/PR/8/34 was grown for 3 days in the allantoic cavity of 10-day embryonated chicken eggs. To remove cellular debris the allantoic fluid was harvested and centrifuged at 3,000 rpm for 30 min. Virus was subsequently purified twice in a discontinuous sucrose gradient at 25,000 rpm for 90 min. Virus stocks were quantified by tissue culture infective dose assay (TCID50). To be inactivated, viral suspensions were placed under the UV lamp at a distance of 15 cm for 15 min. For the labeling of UV-inactivated influenza virus, 50 mg/ml of DiD or DiO dye was added to the viral suspension and incubated for 20 min at RT. After that, virus was subsequently purified by centrifugation as mentioned before.

### Antigen Administration and Injections

10^6^ plaque-forming units (PFU) of inactivated virus per footpad in a final volume of 10 μL were injected into anesthetized mice at different time points prior to LN collection. Macrophage depletion from CD169DTR mice was established by intraperitoneal (i.p.) injection of 10 μg/kg of diphtheria toxin (Sigma-Aldrich) a day before vaccination. Recombinant murine IL-1α (1 μg/fp) and murine CXCL1 (0.5 μg/fp) were reconstituted in sterilized PBS and injected in a final volume of 10 μl 12 h before LN collection. For *in vivo* labeling of cells, mice received subcutaneous injection of 1 μg of fluorescently-labeled αCD21/35, αF4/80, and αCD169/footpad (Biolegend), 3 h before image acquisition. To label the blood vessel mice were injected intravenously with 70 kDa Rhodamin B isothiocyanate-Dextran or Fluorescein isothiocyanate-Dextran solution as described before (97).

### Flow Cytometry

Neutrophil influx to the PLN was monitored using flow cytometry. PLN were collected, disrupted with tweezers, and digested for 10 min at 37°C in an enzyme mix composed of DNase I (0.28 mg/ml, Amresco), dispase (1 U/mL, Corning), and collagenase P (0.5 mg/mL, Roche) in calcium- and magnesium-free PBS (PBS-) followed by a stop solution composed of 2 mM EDTA (Sigma-Aldrich) and 2% heat-inactivated filter-sterilized fetal calf serum (Thermo Fisher Scientific) in PBS- (Sigma-Aldrich). Fc receptors were blocked (αCD16/32, Biolegend) followed by surface staining and analyzed by flow cytometry on a LSRFortessaTM (BD Biosciences). Dead cells were excluded using ZombieAcqua fixable viability dye (Biolegend) and data were analyzed using FlowJo software (TriStar Inc).

### Antibodies

In this study, cell suspension was isolated from harvested organs and immunostained with various combinations of the following fluorescence-conjugated antibodies: αB220 (RA3-6B2), αCD3 (17A2), αCD11b (M1/70), αCD69 (H1.2F3), αI-A/I-E (M5/114.15.2), αLy-6G (1A8), αCD21/CD35 (7E9), αF4/80 (BM8), αCD169 (3D6.112), αCD16/32 (90) (all from Biolegend).

### Cytoplex Assay

The concentration of various cytokines and chemokine in the lymph was determined by LEGENDPlex assays (Mouse Proinflammatory Chemokine Panel and Mouse Inflammation Panel; Biolegend) according to manufacturer's instructions. Briefly, popliteal PLNs were collected and carefully disrupted in 75 μL ice-cold phosphate buffer, minimizing cell rupture. The suspension was centrifuged at 1,500 rpm for 5 min, and the supernatant was collected. Twenty-five microliter supernatant was used for the protocol following the manufacturer instructions. Samples were analyzed by flow cytometry on an LSRFortessa (BD Biosciences), and data were analyzed using LEGENDPlex software (BioLegend).

### Immunohistology and Microscopy

Mice were euthanized, PLN harvested and fixed in 4% PFA at 4°C for 4–6 h. Organs were embedded in 4% low gelling agarose (Sigma-Aldrich) and 50 μm sections were cut with Leica VT1200S vibratome (Leica Microsystems), blocked with proper sera and stained with the indicated antibodies in 0.05% Tween-20 in 0.5% BSA PBS- for two days at 4°C shaking. More details are reported in the antibodies section. Immunofluorescence confocal microscopy was performed using a Leica TCS SP5 confocal microscope (Leica Microsystems). Micrographs were acquired in sequential scans and merged to obtain a multicolor image. Images were processed using Imaris software (Bitplane AG).

### Electron Microscopy

PLN were collected and fixed in 2% formaldehyde 2.5% glutaraldehyde in 0.1 M sodium cacodylate buffer (pH 7.4) overnight at 4°C. LN were washed in 0.05 M maleate buffer (pH 5.15) and stained for 2 h in 1% uranyl acetate in maleate buffer. The samples were dehydrated by incubation for 15 min in ethanol water (60, 90, 100%) and embedded in Epon.

### Intravital Two-Photon Microscopy

Deep tissue imaging was performed on a customized up-right two-photon platform (TrimScope, LaVision BioTec). Two-photon probe excitation and tissue second-harmonic generation (SHG) were obtained with a set of two tunable Ti:sapphire lasers (Chamaleon Ultra I, Chamaleon Ultra II, Coherent) and an optical parametric oscillator that emits in the range of 1,010–1,340 nm (Chamaleon Compact OPO, Coherent), with output wavelength in the range of 690–1,080 nm.

Imaging was performed in the PLN as previously described (98).

### Image Analysis and Data Processing

Cell detection, tracking and volumetric reconstruction from 4D 2P-IVM data were performed using Imaris (Oxford Instruments, v7.7.2). Raw data generated from Imaris were further processed and analyzed with a custom Matlab script.

Cell tracks were generated semi-automatically and curated to correct errors (i.e., jumps or non-detected cells). Tracks with a duration <5 points or 300 s were excluded from the analysis. Videos were stabilized using the drift correction functionality when needed, compensating for translational-drift only and by cropping the largest common area in the videos. Standard measures of cell motility were computed using Imaris. These include: Track duration (time interval between the first and the last time points in which a cell is tracked), Track Length (total length of the cell trajectory), Track Speed Mean (Track length/Track duration), Track Displacement (length of the vector from the first to the latest centroid position of the cell), Track Straightness (Track Displacement/Track Length), and Speed (instantaneous speed computed between adjacent time points).

#### Spectral Unmixing

An additional imaging channel, specific for the cells of interest was generated by classifying each pixel as foreground or background. This was achieved using the Coloc functionality of Imaris in combination with a custom supervised machine learning method for pixel classification implemented in Matlab as described by Pizzagalli and colleagues (99). This method trains a Supported Vector Machine (SVM) to classify pixels as background or background on the basis of examples provided by the user. A minimum of 20 and a maximum of 60 training points were provided for each video. To differentiate between background and foreground the following features were used: Local color, Gaussian-weighted average color in a neighborhood (sigma = 3, 7 μm).

#### Quantification of Cell Density in High Endothelial Venules (HEV) and Lymphatic Vessels (LV)

The density of neutrophils in a vessel presented in Figure 1 is defined as the ratio of the number of cells inside a vessel and the volume of the vessel itself. The density of neutrophils in HEV was computed at different time points inside a selected HEV which was visible for the entire duration of the acquisition (3 h). HEV were labeled by the i.v. injection of 70 kDa Rhodamine B isothiocyanate-Dextran. Cells inside the HEV were manually counted every 300 s. The volume of the HEV was estimated as the volume of a cylinder, by measuring the average diameter in the xy plane and the average height along z and the length of the vessel.

The density of neutrophils in LV at the injection site were computed in a LV visible for the entire duration of the acquisition (4 h). Draining LV were labeled by the subcutaneous injection of DiD-labeled virus and 70 kDa Rhodamine B isothiocyanate-Dextran. Cells were detected and counted automatically using the Spots function of Imaris. The volume of the LV was estimated via volumetric reconstruction using the Surfaces tool of Imaris.

#### Estimation of Distance of the Virus Particle With Respect to Cell Centroid

To confirm the internalization of the viral particles by neutrophils, the distance of the particles from the cell centroid was computed and compared to the cell radius. Cell radius was estimated via volumetric reconstruction of the cell surface and computed as the radius of a sphere having the same volume of the cell.

#### Pixel Velocity

The average velocity of pixels presented in Figure 6A was estimated via a custom Matlab script that computes optical flow as described in Karlsson and Bigun (60). Only the channel where the neutrophils were visible was used for optical flow estimation. To address the lack of texture of immune cells which leads to an aperture problem, Tikhonov regularization was used while computing the flow (Tikhonov constant = 40). Additionally, outliers were removed by Gaussian smoothing (sigma = 7) followed by the saturation of the bottom 1% and the top 1% of all pixel velocity values (set to the minimum and maximum values, respectively).

### Action Recognition

#### Gating Strategy

We mapped a biological meaning (actions) to specific ranges of instantaneous motility measures. This allowed both to describe the dynamic behavior of neutrophils as a series of actions and provided a suggestion on the biological function of each neutrophil at different time points. However, the problem of identifying one action from a range of measures (gate) is an ill-posed problem which often arises when characterizing a complex biological system from experimental data (100). Indeed, not all the possible actions are known and several actions might exhibit similar motility measures leading to an undetermined solution. To solve this issue, we defined gating thresholds that minimize the overlap between distinct actions and we selected one action out of the multiple possible solutions, based on an arbitrary priority order. Although the gates used to detect actions are subjected to the bias of the investigator, these can be easily adapted according to the experimental settings and based on a priori knowledge.

#### Software Implementation

A custom Matlab script (Supplementary Data File 1) was used to automatically compute instantaneous motility measures and detect actions.

This script decomposes each track into track fragments (tracklets) with a fixed duration of 500 s (approximated to 17 time points with a sampling interval of 30 s). From a track with total duration Td ≥ 17 time points, *K* = (Td – 17) + 1 tracklets were extracted by a sliding window. For the analysis of neutrophils actions in homeostatic conditions and due to the high speed of cells flowing in large blood vessels, the time window was reduced to five time points and the sampling interval of the microscope decreased to 20 s.

Then, the script computes the following measures on each tracklet. Displacement: distance between the initial and the final points of the tracks. Speed: track length/track duration. Straightness: displacement/track length.

Arrest coefficient is typically defined as the percentage of time in which a cell moves below a certain speed threshold. However, being the tracklets of short duration this results in a limited number of admissible percentages. Additionally, a sharp threshold may introduce artifacts. Therefore, we computed arrest coefficient by using a sigmoidal thresholding function defined as follows.

$$\begin{array}{r} \text{ac}=\frac{\sum_{i=1}^{k}1-\frac{1}{e^{1+s\left(k\right)-\tau }}}{\text{a}{\text{c}}_{0}} \end{array}$$

where τ = 2 μm/min is a speed threshold and ac_0_ = k – (k/e^1−τ^) is the arrest coefficient of a cell having a constant speed of 0 μm/min.

Based on the aforementioned parameters, each tracklet was associated with one of the following actions:

“Arrested”: Cell which does not move (i.e. interacting/adhering/death), exhibiting low speed, low directionality, low displacement, and high arrest coefficient.

“Patrolling”: Cell which moves, covering a large area of tissue, with medium speed and directionality.

“Directed”: Cell which moves toward a target exhibiting high speed and directionality (high displacement and low arrest coefficient).

“Flowing”: Cell which flows inside the capillaries of blood vessels in the LN. A flowing cell exhibits extremely high speed and directionality.

Tracklet-to-action association was implemented by means of fixed thresholds corresponding to different gates defined in Table 1.

**Table 1:** Gating thresholds.

**Evaluated on 500 s**		**Arrested**	**Patrolling**	**Directed**	**Flowing**	**Swarming**
Speed (μm/min)	Min	0	2	6	10	–
	Max	7	8	25	300	–
Straightness (0.1)	Min	0	0.1	0.3	0.5	–
	Max	0.3	0.5	1	1	–
Arrest coefficient (0.1)	Min	0.7	0.1	0	0	–
	Max	1	0.8	0.2	0.2	–
Displacement (0.1)	Min	0	4	8	50	–
	Max	15	20	80	300	–
Volume (μm^3^)	Min	–	–	–	–	2,000

*Each action (row) is mapped to a specific range (min-max) of motility parameters (columns). Values refer to tracklets with a duration of 500 s*.

The detection of cells involved in a swarm (swarming) was achieved via a volumetric reconstruction. For the videos in Figure 4 with adoptively transferred CK6/ECFP neutrophils, swarms were considered as surfaces with a minimum volume of 2^*^10^3^ μm^3^.

The number of cells in a swarm was estimated by dividing the swarm volume by the volume of a single cell (assumed to 1^*^10^3^ μm^3^ without spaces between cells).

### Swarm Quantification

To quantify the size and the growth rate of swarms we defined both a cell density and an overall directionality criteria. More precisely, we detected swarms in regions where cells accumulated and where most trajectories pointed to. The “surfaces” functionality of Imaris was used to reconstruct the volume of swarms, selecting the marching cube algorithm with either a user-defined brightness threshold in low-magnification videos–Figure 6C or by manually defining a region of interest around the swarm in high magnification videos–Figure 6I. Neutrophils involved in the swarm were manually tracked until the end of the videos or when they merged to emerging swarms.

### Statistics

Results were expressed as mean ± standard deviation (SD). All statistical analyses were performed in Prism8 (Graphpad Software, La Jolla, USA). Means among two groups were compared with two-tailed *t*-test. Means among three or more groups were compared with one-way ANOVA with Dunn's multiple comparison post-test.

### Software Availability and Usage

The source code of the program to quantify the actions of immune cells from their tracks, is provided in Supplementary Data File 1. This program requires the tracks of the cells to be exported from Imaris to an Excel file. After this has been done, it is possible to open the program in Matlab, enter the location of the Excel file, and executing the program. The plots counting the actions will be automatically created.

To facilitate this process, and to avoid the requirement of Matlab, the software will be further distributed as an open source plug-in for common imaging software at https://www.ltdb.info/tool and https://github.com/IRB-LTDB/.

## Data Availability Statement

The datasets generated for this study are available on request to the corresponding author.

## Ethics Statement

All animal experiments were performed in accordance with the Swiss Federal Veterinary Office guidelines and authorized by the relevant institutional committee (Commissione cantonale per gli esperimenti sugli animali, Ticino) of the Cantonal Veterinary with authorization numbers TI28/17, TI02/14, and TI07/13.

## Author Contributions

SG directed the study. SG, DP, IL, and AP designed and performed experiments, analyzed and interpreted the results, and wrote the manuscript. RK supervised the computational aspects of data analysis. SG and MP-S performed 2P-IVM. YF and TV performed experiments.

### Conflict of Interest

The authors declare that the research was conducted in the absence of any commercial or financial relationships that could be construed as a potential conflict of interest.

## Supplementary Material

Supplementary Movie 1 Recruitment of neutrophils from blood vessels. 4D 2P-IVM video acquired in the LN showing LysM-GFP neutrophils (green) recruited via blood vessels (labeled with i.v. injection of Rhodamin B isothiocyanate-Dextran–red, dashed line). Arrow indicates a hotspot where neutrophils accumulate during extravasation.

Supplementary Movie 2 Recruitment of neutrophils from lymphatic vessels. 4D 2P-IVM video acquired in the site of injection, showing LysM-GFP neutrophils (green) recruited via lymphatic vessels (red) that draining to lymph node. UV-PR8 is labeled in FR (white) and collagen fibers are visible by second harmonic generation (SHG, blue).

Supplementary Movie 3 Neutrophils phagocytize influenza virus particles. 4D 2P-IVM video acquired in the LN showing an interaction between LysM-GFP neutrophils (green) and viral aggregates (UV-PR8-DiO, red). Lines indicate the tracks of neutrophils and viral aggregates, respectively.

Supplementary Movie 4 Migration of neutrophils in the LN at 30, 75, and 135 min post-vaccination. Neutrophils (light-blue) adoptively transferred from a CK6/ECFP animal, migrate in the lymph node exhibiting different motility patterns over time. The vascular network is labeled via i.v. injection of Fluorescein isothiocyanate-Dextran (green), macrophages are labeled via CD169-PE antibody (red) while the collagen scaffold is visible via Second Harmonic Generation (SHG, blue).

Supplementary Movie 5 Neutrophil migration in different areas of the LN. 4D 2P-IVM video acquired using a low magnification (10x) showing the migration of LysM-GFP neutrophils (green) in the different areas of the LN. Collagen scaffold is visible via Second Harmonic Generation (SHG, blue).

Supplementary Movie 6 Swarm formation after vaccination. 4D 2P-IVM video showing the formation of multiple LysM-GFP neutrophils swarms (green) in the SCS in close proximity to CD169-AF647 macrophages (white). Blood vessels are labeled via i.v. dextran injection (red) and the LN scaffold is visible via Second Harmonic Generation (SHG, blue).

Supplementary Movie 7 Swarm formation associated with dying macrophages. 4D 2P-IVM video showing swarm formation after vaccination. CFP-expressing neutrophils (light-blue) are adoptive transfer into CD11c-YFP (green) animal prior vaccine injection. UV-PR8 is labeled with DiD labeling dye (red). Dashed line indicates the subcapsular sinus area and arrows indicate recruited neutrophils.

Supplementary Movie 8 Neutrophil migration in homeostasis. Neutrophils (light-blue) adoptively transferred from a CK6/ECFP animal flow within the blood vessels of the lymph node. Vascular network (labeled via i.v. injection of Fluorescein isothiocyanate-Dextran (green), macrophages are labeled via CD169-PE antibody (red) while the collagen scaffold is visible via Second Harmonic Generation (SHG, blue).

Supplementary Figure 1 **(A)** Scanning electron micrograph showing an association between neutrophils (n) and HEV at 2 h post vaccination (p.v.). The lumen of the blood vessel is marked in red. **(B)** Flow cytometric analysis showing the recruitment of neutrophils (Ly6G+ CD11b+) at 12 h p.v. Percentages of CD44+ **(C)**, CD62L **(D)** and CD49d+ **(E)** cells out of all neutrophils at 12 h p.v. compared with non-vaccinated controls. **(F)** Absolute numbers of CD169+ cells in the lymph node of DT treated CD169-DTR mice (left) and representative density dot plot showing the gating for CD169+CD11c intlow macrophage population (right). Quantification of CD169+ macrophages in CCR2KO **(G)** and IFNARKO mice **(H)**. **(I)** Total number of CD169+F4/80+ medullary macrophages in IFNARKO mice. **(J)** Schematic drawing (left) of an electron micrograph (right) showing neutrophils in the SCS phagocytizing UV-inactivated influenza virus at 2 h p.v.

Supplementary Figure 2 **(A)** 2P-IVM micrograph showing the vascular structure of the popliteal lymph node and the tracks of neutrophils (white lines) prior to vaccination. Only neutrophils visible for at least 100 s and migrating within the lymph node are tracked. **(B,C)** Actions performed by the tracked neutrophils corresponding to distinct motility values shown in **(B)** mainly associated to flowing **(C)**.
