# Supplementary figures and images for: Characterization of the Dynamic Behavior of Neutrophils Following Influenza Vaccination
Source: Front Immunol. 2019 Nov 20;10:2621. doi: 10.3389/fimmu.2019.02621 (PMC6881817; doi:10.3389/fimmu.2019.02621)

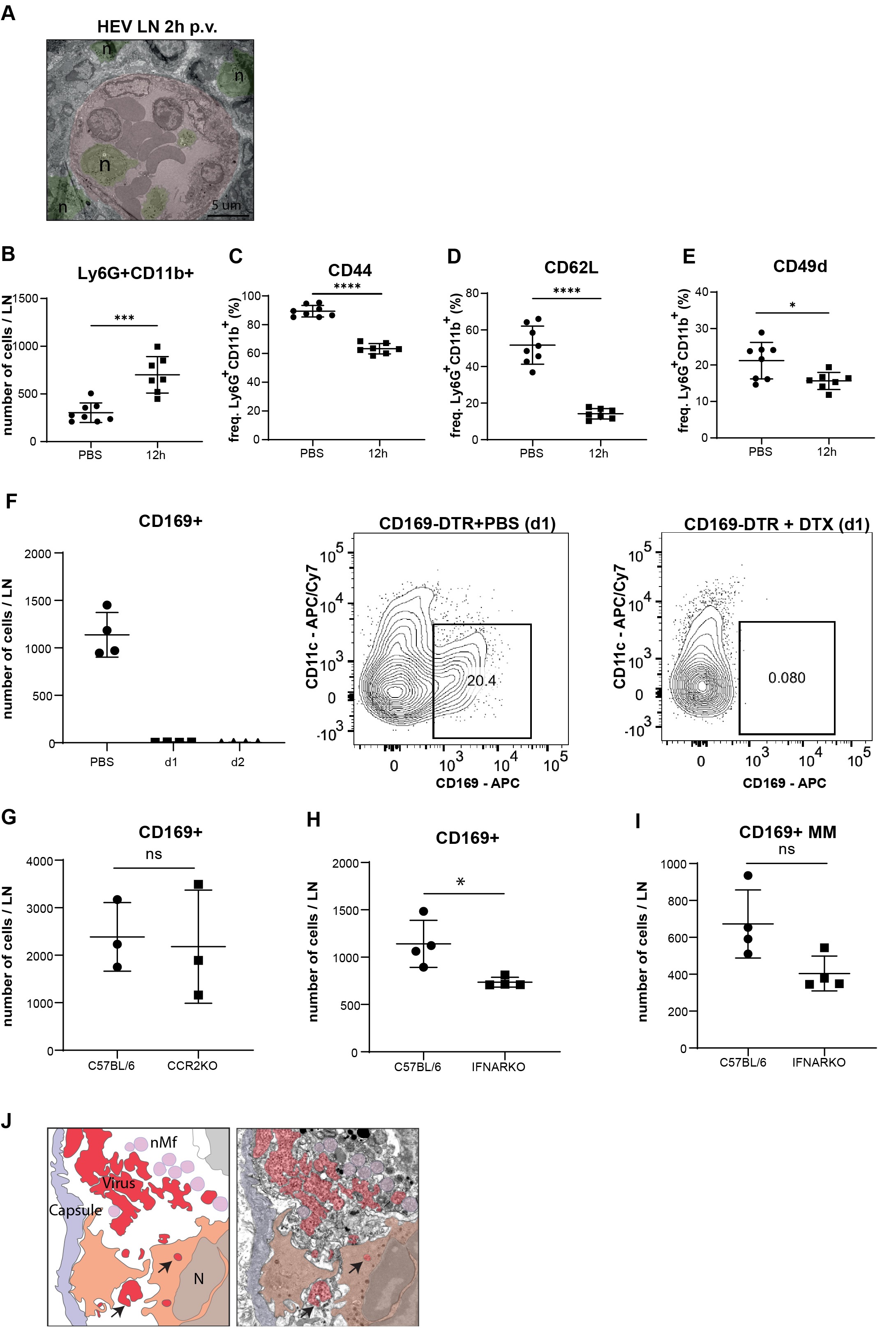

Supplement: Supplementary Figure 1 — (A) Scanning electron micrograph showing an association between neutrophils (n) and HEV at 2 h post vaccination (p.v.). The lumen of the blood vessel is marked in red. (B) Flow cytometric analysis showing the recruitment of neutrophils (Ly6G+ CD11b+) at 12 h p.v. Percentages of CD44+ (C), CD62L (D) and CD49d+ (E) cells out of all neutrophils at 12 h p.v. compared with non-vaccinated controls. (F) Absolute numbers of CD169+ cells in the lymph node of DT treated CD169-DTR mice (left) and representative density dot plot showing the gating for CD169+CD11c intlow macrophage population (right). Quantification of CD169+ macrophages in CCR2KO (G) and IFNARKO mice (H). (I) Total number of CD169+F4/80+ medullary macrophages in IFNARKO mice. (J) Schematic drawing (left) of an electron micrograph (right) showing neutrophils in the SCS phagocytizing UV-inactivated influenza virus at 2 h p.v. [file Image_1.jpg]

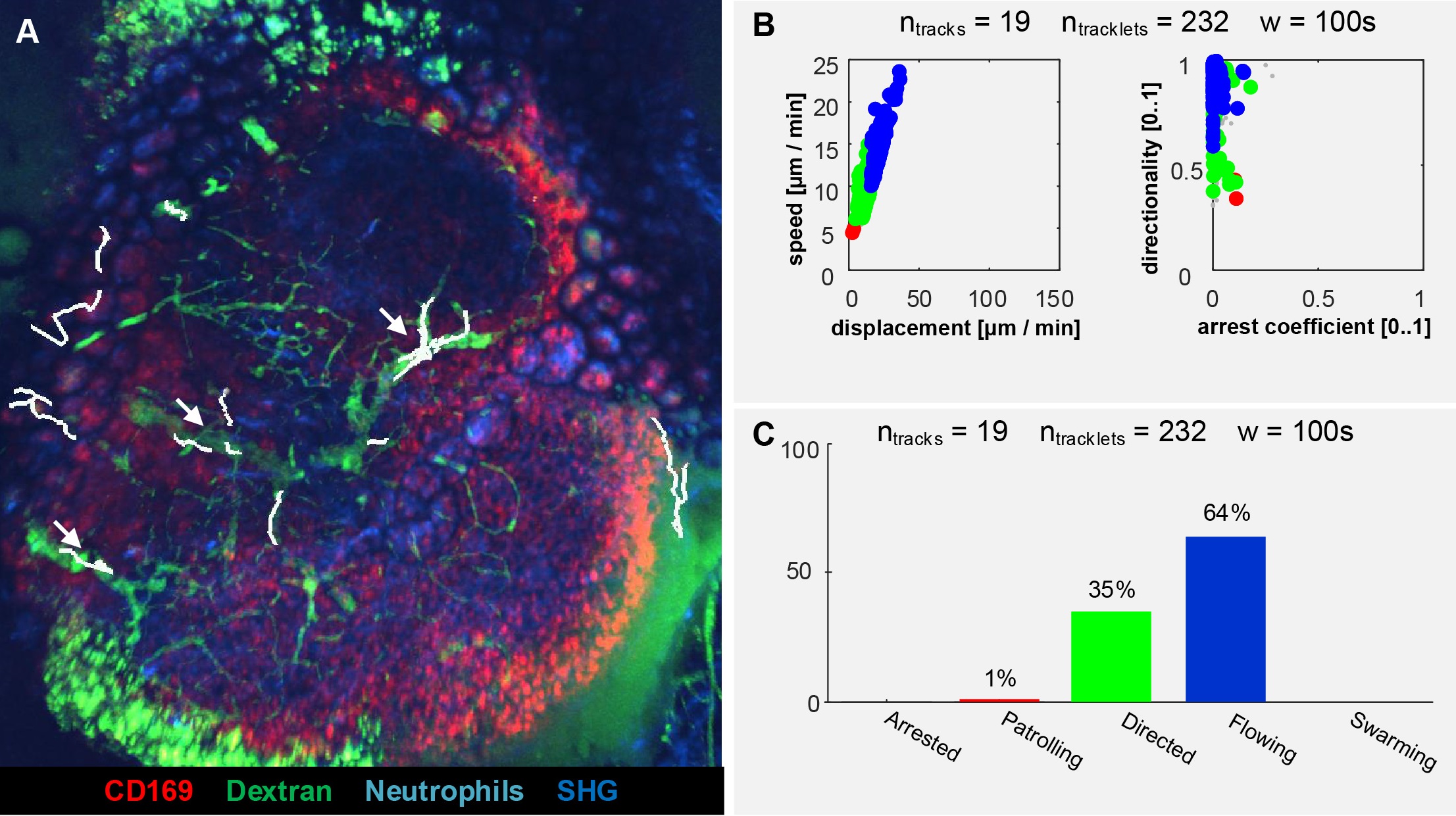

Supplement: Supplementary Figure 2 — (A) 2P-IVM micrograph showing the vascular structure of the popliteal lymph node and the tracks of neutrophils (white lines) prior to vaccination. Only neutrophils visible for at least 100 s and migrating within the lymph node are tracked. (B,C) Actions performed by the tracked neutrophils corresponding to distinct motility values shown in (B) mainly associated to flowing (C). [file Image_2.jpg]
